# Detection of large molecular weight cytokeratin 8 as carrier protein of CA19–9 in non-small-cell lung cancer cell lines

**DOI:** 10.1038/sj.bjc.6690762

**Published:** 1999-11

**Authors:** J Fujita, N Dobashi, Y Ohtsuki, Y Ueda, S Bandoh, I Yamadori, J Takahara

**Affiliations:** First Department of Internal Medicine, Kagawa Medical University, 1750-1, Miki-cho, Kita-gun, Kagawa, 761-0793, Japan; Department of Pathology, Kochi Medical School, Kochi, Japan; Department of Pathology, Okayama University Medical School, Okayama, Japan

**Keywords:** antibody, CA19–9, cytokeratin 8, non-small-cell lung cancer

## Abstract

It has been reported that cytokeratin 8 (CK8) is expressed in all non-small-cell lung cancers (NSCLC). We hypothesized that antigenic changes of CK8 may occur in some NSCLC cell lines. To prove this, Western immunoblot analysis using anti-human CK8 monoclonal antibodies as well as immunohistological staining of CK8 were performed in NSCLC cell lines. As a result, CK8 which had a higher molecular weight than recombinant CK8 was demonstrated in two of eight NSCLC cell lines. In addition, this CK8 contained antigenic epitopes of CA19–9. This CK8 with higher molecular weight, may have played a role in the process of invasion or metastasis of NSCLC. © 1999 Cancer Research Campaign


British Journal of Cancer (1999) 81(5), 769–773
© 1999 Cancer Research Campaign
Article no. bjoc.1999.0762

### Detection of large molecular weight cytokeratin 8 as carrier protein of CA19–9 in non-small-cell lung cancer cell lines

**J Fujita[1], N Dobashi[1], Y Ohtsuki[2], Y Ueda[1], S Bandoh[1], I Yamadori[3] and J Takahara[1]**

[1]First Department of Internal Medicine, Kagawa Medical University, 1750-1, Miki-cho, Kita-gun, Kagawa, 761-0793, Japan; [2]Department of Pathology, Kochi Medical School, Kochi, Japan; [3]Department of Pathology, Okayama University Medical School, Okayama, Japan

**Summary** It has been reported that cytokeratin 8 (CK8) is expressed in all non-small-cell lung cancers (NSCLC). We hypothesized that antigenic changes of CK8 may occur in some NSCLC cell lines. To prove this, Western immunoblot analysis using anti-human CK8 monoclonal antibodies as well as immunohistological staining of CK8 were performed in NSCLC cell lines. As a result, CK8 which had a higher molecular weight than recombinant CK8 was demonstrated in two of eight NSCLC cell lines. In addition, this CK8 contained antigenic epitopes of CA19–9. This CK8 with higher molecular weight, may have played a role in the process of invasion or metastasis of NSCLC.
© 1999 Cancer Research Campaign

**Keywords:** antibody; CA19–9; cytokeratin 8; non-small-cell lung cancer

Cytokeratin 8 (CK8) is one of at least 21 related cytokeratins that form intermediate filaments in various epithelial cells and carcinoma cells (Moll et al, 1982). Numerous studies have shown that high levels of CK8 exist in malignant cells. It has been reported that CK8 can be expressed at the surface of mammary carcinoma cells but not in normal mammary epithelial cells (Donald et al, 1991; Godfroid et al, 1991; Hembrough et al, 1995, 1996). Similarly, it has been shown that all non-small-cell lung cancers (NSCLC) express CK8 (Blobel et al, 1984; Broers et al, 1988; Pendleton et al, 1992, 1994).

It has also been reported that the expression of CK8 has been correlated with increased invasiveness in vitro and in vivo. In malignant melanoma, in vitro invasiveness has been directly correlated with cellular expression of intermediate filaments (Hendrix et al, 1996). In transitional cell carcinoma and squamous cell carcinoma, CK8 has been detected at increased levels by immunohistochemistry at the tumour invasion front (Schaafsma et al, 1991, 1993). In addition, the most tumorigenic clones of SW 613-S cells express the highest levels of CK8 mRNA (Modjtahedi et al, 1992). Furthermore, mouse L fibroblasts, which lack CK8 and 18, show increased motility and penetration of Matrigel in vitro after transfection of CK8 DNA (Chu et al, 1993).

CK8 or CK8 fragments have also been detected in conditioned culture medium (Chan et al, 1986) and in the serum and body fluids of patients with a variety of cancer types (Bjorklund and Bjorklund, 1983; Pendleton et al, 1994). One study has demonstrated the presence of elevated levels of CK8 and degraded fragments of this polypeptide in patients with colonic and pancreatic tumours (Sundstrom et al, 1990). In addition, Pendleton et al (1994) have demonstrated that undegraded CK8 is found in the serum of a subgroup of patients with NSCLC. Furthermore, autoantibodies to intermediate filaments have been found in patients with lung cancer (Hinter et al, 1983).

Under this background, we hypothesized that some antigenic changes of CK8 may occur in human NSCLC. To prove this, a Western immunoblot analysis and immunohistochemistry of CK8 was performed in NSCLC cell lines.

*Received 24 November 1998*
*Revised 28 April 1999*
*Accepted 10 May 1999*

*Correspondence to:* J Fujita

### MATERIALS AND METHODS

### Cell lines (Table 1)

We used cell lines as follows: A549, PC3, PC9, RERF-LC-OK, LC2/AD (derived from adenocarcinoma of the lung), EBC1, VMRC-LCD and LC1/SQ (derived from squamous cell carcinoma of the lung). All cell lines were cultured in RPMI-1640 with 10% fetal calf serum.

### SDS-PAGE electrophoresis and Western blotting

Sodium dodecyl sulphate polyacrylamide gel electrophoresis (SDS-PAGE) was performed according to Laemmli's method (Laemmli, 1970) with a slight modification. Whole cell lysates of cell lines were mixed with SDS (2.0%) and heated (100°C, 5 min). The samples were then applied to a 10/20% SDS polyacrylamide gel, electrophoresed (60 mA, 120 min), fixed in 50% methanol, 10% acetic acid and stained with Coomassie Blue. Standard molecular weight markers purchased from Daiichi-Kagaku (Tokyo, Japan) were comprised of egg lysozyme (MW 14 400), trypsin inhibitor (MW 20 100), carbonic anhydrase (MW 30 000) aldolase (MW 42 400) bovine albumin (MW 66 000) and phosphorylase B from rabbit muscle (MW 97 400). Commercially available purified recombinant human CK8 (Progen Biotechnik GMBH, lot 503209, Heiderberg, Germany) was also used as the positive control.

**Table 1** Lung cancer cell lines evaluated

| Cell lines | Histology | Western immunoblot | Immunohistochemistry |
|---|---|---|---|
| A549 | Adenocarcinoma | + | + |
| PC3 | Adenocarcinoma | + | + |
| PC9 | Adenocarcinoma | + | + |
| RERF-LC-OK | Adenocarcinoma | +[a] | –[b] |
| LC2/AD | Adenocarcinoma | + | + |
| EBC1 | Squamous cell carcinoma | + | + |
| VMRC-LCD | Squamous cell carcinoma | ± | + |
| LC1/SQ | Squamous cell carcinoma | +[a] | –[b] |

[a]Cytokeratin 8 which had a higher molecular weight. [b]Clone 35βH11 was used as anti-cytokeratin 8 antibody.

Proteins were electrophoretically transferred onto nitrocellulose membrane by the method of Towbin et al (1983). Proteins were detected by immunoblotting, using five clones of anti-CK8 monoclonal antibodies (clones Ks 8.7, Ks 8.10, Ks 8.17.2, and M20 were purchased from Progen Biotechnik GMBH, Heidelberg, Germany; clone C-51 was purchased from YLEM S. r. 1., Roma, Italy), peroxidase-conjugated goat anti-mouse IgG antibody (Sigma ImmunoChemicals, lot 094H-4810, St Louis, MO, USA), and stained with 4CN PLUS for chromogenic detection of horseradish peroxidase (NEM$^{TM}$ Life Science Products, Boston, MA, USA). Western immunoblots for CA19–9 were also performed using three clones of anti-CA19–9 monoclonal antibodies (clone C241:5:1:4 was purchased from YLEM S. r. 1., Roma, Italy; clone ZY-CO9 was purchased from Zymed Laboratories, Inc., San Francisco, CA, USA; clone 1116–NS–19–9 was purchased from Centocor Co., Malvern, PA, USA), and peroxidase-conjugated goat anti-mouse IgG antibody IgG antibody, and stained with 4CN PLUS.

### Immunohistochemistry of lung cancer cells by several anti-CK8 antibodies

To evaluate the expression of CK8 in several NSCLC cell lines, immunohistochemical staining by anti-human monoclonal antibody against CK8 was performed. Cells were immunohistochemically stained, employing the avidin–biotin peroxidase complex method (Dako LSAB kit-peroxidase, DAKO Corp., Kyoto, Japan) using mouse monoclonal antibodies against CK8 (clone 35 βH11, Enzo Diagnostics, Inc., New York, NY, USA, 1:5000 dilution). In order to retrieve and increase the immunoreactivities, preincubation with 0.1% pronase at 37°C for 20 min was performed.

### Lectin blotting

SDS-PAGE and protein transfer onto nitrocellulose membrane was performed as described above. Ten kinds of horseradish peroxidase-labelled lectins, purchased from EY Laboratories, Inc. (San Mateo, CA, USA), were used for lectin blotting. Proteins were detected by ten kinds of peroxidase-labelled lectins (1:100 dilutions, following manufacturer's instruction), and stained with 4CN PLUS. Lectins used were as follows; *Maclura pomifera* lectin (MPA), *Aracjis hypogaca* lectin (PNA), *Glycine max* lectin (SBA), *Griffonia simplicifolia* lectin (GS-II), *Griffonia simplicifolia* lectin (GS-I), *Ulex europaeus* lectin (UAE-I), *Triticum vulgare* lectin (WGA), *Dolichos biflorus* lectin (DBA), *Canavalia ensiformis* lectin (Con A) and *Bauhinia purpurea* lectin (BPA).

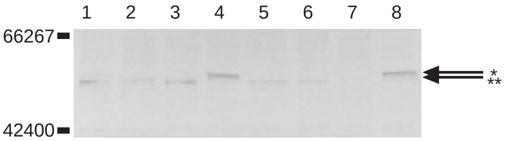

**Figure 1** Western immunoblot analysis using anti-cytokeratin 8 monoclonal antibody (clone Ks 8.7) against lysates of several NSCLC cell lines. Lane 1; A549, lane 2; PC3, lane 3; PC9, lane 4; RERF-LC-OK, lane 5; LC2/AD, lane 6; EBC1, lane 7; VMRC-LCD, lane 8; LC1/SQ. Although 54 kDa cytokeratin 8 is demonstrated in A549, PC3, PC9, LC2/AD (arrow *), another band which has a higher molecular weight than usual is demonstrated in RERF-LC-OK and LC1/SQ (arrow **)

### RESULTS

Western immunoblot analysis using anti-CK8 antibody (clone Ks 8.7) against lysates of several NSCLC cell lines is shown in Figure 1. Although a molecular weight of 54 kDa, which is the same as recombinant CK8, was detected in PC3, PC9, LC2/AD and EBC1, a positive band which had a higher molecular weight was detected in RERF-LC-OK and LC1/SQ cells. In A549 cell lines, two types of CK8 were observed.

Western immunoblot analysis using several monoclonal anti-CK8 antibodies demonstrated that the higher molecular weight protein was stained by five clones of anti-CK8 antibodies with varying intensities (Figure 2). Although CK8 with a higher molecular weight was weakly stained by monoclonal antibodies; clones Ks 8.7, Ks 8.10 and M20, it was strongly stained by monoclonal anti-CK8 antibodies; clone Ks 8.17.2 and clone C-51.

Western immunoblot analysis using several monoclonal anti-human CA19–9 antibodies also demonstrated that the higher molecular weight protein was stained by three clones of anti-human CA19–9 antibodies (Figure 3). Recombinant CK8 was also weakly stained by anti-human CA19–9 antibodies.

Immunohistochemical staining of several lung cancer cells with anti-CK8 monoclonal antibody was restricted to the cytoplasm, and the cell membranes were completely negative (Figure 4). The two cell lines containing CK8 with a higher molecular weight (RERF-LC-OK and LC1/SQ) were not stained by anti-CK8 antibody; clone 35βH11. Table 1 summarizes the results of immunohistochemical staining of several NSCLC cell lines.

Results of lectin blotting using ten kinds of peroxidase-labelled lectins are summarized in Table 2. The higher molecular weight CK8 was stained stronger than usual CK8 by MPA, GS-II, UEA-I, DBA and BPA lectins.

 © 1999 Cancer Research Campaign

**Table 2** Summary of results of lectin blotting

| Lectins | Carbohydrate specificity | CK8 | Large CK8 |
|---|---|---|---|
| MPA | N-Acetylgalactosamine | ± | ++ |
| PNA | Terminal-β-Galactose | – | ± |
| SBA | α and β N-Acetylgalactosamine | – | – |
| GS-II | N-Acetylglucosamine | – | ++ |
| GS-I | Melibiose, α-D-Galactose | – | + |
| UEA-I | α-L-Fucose | ± | ++ |
| WGA | (GlcNAc-β-(1,4)-GlcNAc) 1–4 | + | + |
| DBA | Methyl-2-acetamido-2-deoxy-D-galactose | + | ++ |
| Con A | α-D-Mannose, α-D-Glucose, Branched mannose | – | – |
| BPA | N-Acetylgalactosamine | – | ++ |

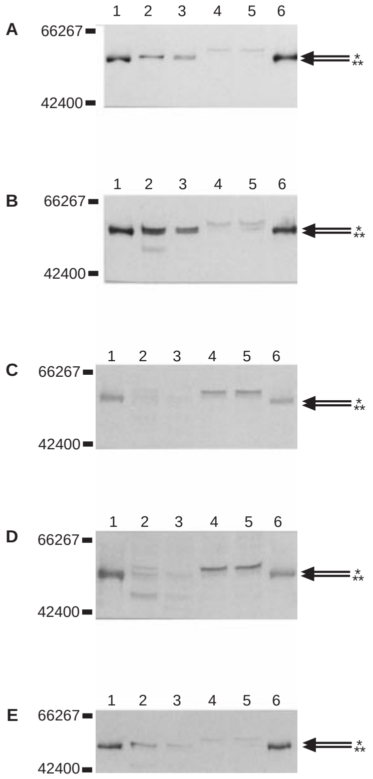

**Figure 2** Western immunoblot analysis using the five clones of anti-human cytokeratin 8 monoclonal antibodies. (**A**) clone Ks 8.7, (**B**) clone Ks 8.10, (**C**) clone Ks 8.17.2, (**D**) clone C-51, (**E**) clone M20. Lane 1 and 6: recombinant cytokeratin 8, lane 2: A549, lane 3: PC9, lane 4: RERF-LC-OK, lane 5: LC1/SQ. Although 54 kDa cytokeratin 8 is demonstrated in A549, PC3, PC9, LC2/AD (arrow *), another band which has a higher than usual molecular weight is demonstrated in RERF-LC-OK and LC1/SQ (arrow **). The intensity of staining for CK8 with a higher than usual molecular weight differs according to clones of antibodies

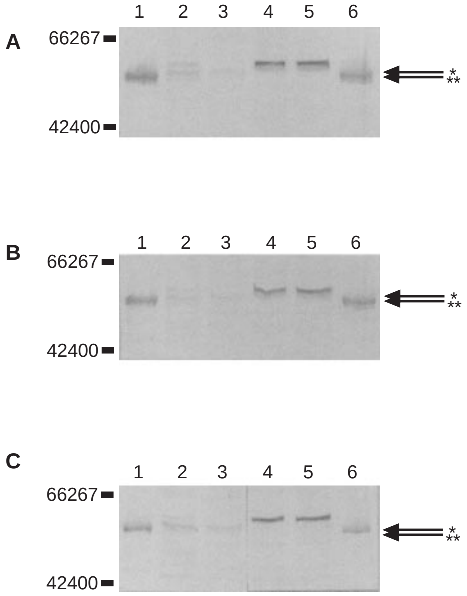

**Figure 3** Western immunoblot analysis using the three clones of anti-human CA19-9 monoclonal antibodies. (**A**) clone C 241:5:1:4, (**B**) clone ZY-C09, (**C**) clone 1116-NS-19-9. Lane 1 and 6: recombinant cytokeratin 8, lane 2: A549, lane 3: PC9, lane 4: RERF-LC-OK, lane 5: LC1/SQ. Although 54 kDa cytokeratin 8 (arrow **) in NSCLC cell lines is not stained by anti-CA19-9 monoclonal antibodies, cytokeratin 8 which has a higher than usual molecular weight (arrow*) is clearly stained by all anti-CA19-9 monoclonal antibodies. Recombinant cytokeratin 8 is also weakly stained by anti-CA19-9 monoclonal antibodies

### DISCUSSION

In our present study, we confirmed the expression of CK8 in all NSCLC cell lines. In addition, we first demonstrated CK8 with a higher molecular weight than usual in two of eight NSCLC cell lines, and it contained antigenic epitopes of CA19–9.

It has been reported that CK8 extends from the nucleus to the plasma membrane where it has extensive interactions with the internal leaflet and with various membrane-associated structures, including desmosomes and hemidesmosomes (Owaribe et al, 1991; Garrod, 1993). Although the sequence of CK8 does not include a transmembrane domain, it has been proposed that CK8 is also present on the external surfaces of epithelial cells (Donald et al, 1991; Godfroid et al, 1991; Hembrough et al, 1995, 1996).

Recently, it has been suggested that CK8 has a function in addition to as an intermediate filament. Schaafsma et al (1991, 1993) have suggested that expression of CK8 correlates with increased invasiveness in vitro and in vivo in many cancers. In addition, Hembrough et al (1995, 1996) reported that CK8 at the cell surface of hepatocytes, HepG2 cells and breast carcinoma cell lines function as a plasminogen receptor. Since plasmin which is activated by the proteolytic conversion of single-chain plasminogen is important for tumour invasion and cellular migration, CK8, which by binding plasminogen, supports or accelerates cellular migration and invasion (Hembrough et al, 1995, 1996).

This is the first study to report CK8 with a higher than usual molecular weight in two NSCLC cell lines (RERF-LC-OK and

*© 1999 Cancer Research Campaign* 

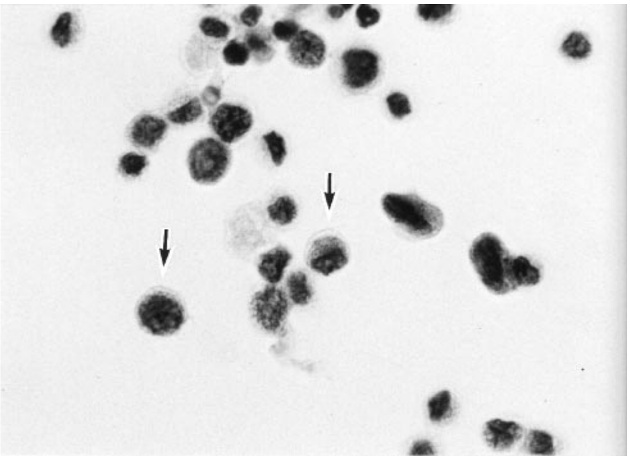

**Figure 4** Immunohistochemical staining of lung cancer cells (PC9 cells) with anti-cytokeratin 8 monoclonal antibody. Positive staining is restricted to the cytoplasm (arrows), and the cell membranes are completely negative. Clone 35βH11 is used as an anti-cytokeratin 8 monoclonal antibody and haematoxylin stain is also performed to stain the nuclei

LC1/SQ). In addition, although it has been reported that CK8 is glycosylated at multiple sites with a single O-linked N-acetyl-glucosamine (Chou et al, 1992), we demonstrate for the first time that CK8 with a higher than usual molecular weight contained antigenic epitopes of CA19–9. In addition, using lectin blotting, we also demonstrated that N-acetylgalactosamine (detected by MPA lectin), N-acetylglucosamine (detected by GS-II lectin), and α-L-fucose (detected by UEA-I lectin), these carbohydrates are constituents of antigenic epitopes of CA19–9, were increased in a higher molecular weight CK8. Furthermore, this CK8 was only weakly stained by some clones of anti-CK8 monoclonal antibodies, and cell lines which expressed CK8 with a higher than usual molecular weight were not stained by anti-CK8 monoclonal antibody; clone 35βH11, immunohistochemically. Interestingly, CK8 was stained only in the cytoplasm in lung cancer cell lines which expressed CK8 with a usual molecular weight. In contrast, the two cell lines containing CK8 with a higher molecular weight (RERF-LC-OK and LC1/SQ) were not stained by anti-CK8 antibody; clone 35βH11. This evidence suggests that antigenic changes of CK8 occurred in both of these NSCLC cell lines. These antigenic changes may explain previous observations of anti-cytokeratin antibodies in sera of patients with lung cancer (Hinter et al, 1983).

Although the assay of serum CA19–9 has been widely used for the diagnosis and monitoring patients with several kinds of cancer, less is known about the protein(s) on which CA19–9 is expressed. Koprowski et al (1979) have revealed that monoclonal antibodies for the CA19–9 antigen precipitates the molecules with molecular weight of 36 kDa and more than 180 kDa from colon carcinoma cell extract. On the other hand, the CA19–9 immunoreactivity detected in the sera of patients (Magnani et al, 1983), normal and neoplastic mucosa (Fezi et al, 1984), normal seminal plasma (Hanisch et al, 1984), normal milk (Hanisch et al, 1985) and pancreatic juice (Kalthoff et al, 1986), has been reported to have a much higher molecular weight probably due to complex formation with other components such as mucin-like glycoproteins. In addition, Klug et al (1988) purified a glycoprotein (an apparent molecular mass of 210 kDa) with the CA19–9 activity from the culture supernatant of human colonic cell line SW1116 by using an immunoaffinity chromatography. Furthermore, Haga et al (1989) have partially purified the CA19–9 antigen from the ascitic fluid of a pancreatic cancer patient, and determined the molecular weight to be 210 kDa by Western blotting analysis. These observations suggest that the CA19–9 epitopes of the cancer cell surface is not expressed by a single glycoprotein but, rather, by multiple glycoproteins. In the present study, we first demonstrate the possibility of CK8 as a carrier protein of CA19–9 in some NSCLC cell lines. The result of lectin blotting clearly demonstrated increases of carbohydrate epitopes in CK8 with a higher molecular weight. However, since the recombinant CK8 was also stained by anti-CA19–9 antibodies, the possibility of cross-reactivity of anti-carbohydrate antibodies to the specific amino acid sequence in recombinant CK8 should also be considered.

The biological significance of CA19–9 should be discussed. Basu et al (1987) reported that a significant portion (20–80%) of protein-associated CA19–9 of human cancer cell membranes is intrinsic to a 170 kDa epidermal growth factor (EGF) receptor. However, Klug et al (1988) reported that the amino acid composition of the glycoprotein which contains the CA19–9 antigen is different from that of the EGF receptor. Recent studies also provide evidence that CA19–9 can serve as a ligand for the endothelial cell leucocyte adhesion molecule-1 (ELAM-1, also called E-selectin) (Takada et al, 1991, 1993). ELAM-1 mediates the cell–cell interaction of platelets and endothelial cells with neutrophils, monocytes and also cancer cells. Thus, when cancer cells express CA19–9, they appear to play an important role in the process of haematogenous metastasis (Takada et al, 1991, 1993). Several immunohistochemical studies have demonstrated that expression of CA19–9 is correlated with a high risk of distant metastasis and poor outcome in lung cancer patients (Sugiyama et al, 1992, Ogawa et al, 1994). This evidence suggests strongly that CA19–9 also plays a role in metastatic processes, in vivo.

As stated previously, CK8 has several functions including the invasion of cancer cells. In addition, CA19–9 plays a role as a ligand of E-selectin which is closely related to metastasis. This evidence suggests that CK8 which contained antigenic epitopes of CA19–9 played an important role in invasion as well as metastasis of NSCLC cells. The existence of this abnormal CK8 in clinical samples should be evaluated in future studies. In addition, to clarify the function of this modified CK8, the differences of CK8 between primary and metastatic tumours should be evaluated in future studies.

In summary, this is the first report of CK8 with a higher than usual molecular weight, possibly due to the attachment of antigenic epitopes of CA19–9, in two of eight NSCLC cell lines. This CK8 may have functions in the process of invasion or haematogeneous metastasis in NSCLC.

### REFERENCES

Basu A, Murthy U, Rodeck U, Herlyn M, Mattes L and Das M (1987) Presence of tumor-associated antigens in epidermal growth factor receptors from different human carcinomas. *Cancer Res* **47**: 2531–2536

Blobel GA, Moll R, Franke WW and Vogt-Moykopf I (1984) Cytokeratins in normal lung and lung carcinomas: adenocarcinomas, squamous cell carcinomas and culture cell lines. *Virchows Arch (Cell Pathol)* **45**: 409–429

Broers JLV, Ramaekers FCS, Klein Rot M, Oostendorp T, Huysmans A, van Muijen GNP, Wagennar S and Vooiji GP (1988) Cytokeratins in different types of human lung cancer as monitored by chain-specific monoclonal antibodies. *Cancer Res* **48**: 3221–3229

 © 1999 Cancer Research Campaign

Bjorklund B and Bjorklund V (1983) Specificity and basis of the tissue polypeptide antigen. *Cancer Detec Prevent* **6**: 411–450

Burchill SA, Bradbury MF, Pittman K, Southgate J, Smith B and Selby P (1995) Detection of epithelial cancer cells in peripheral blood by reverse transcriptase-polymerase chain reaction. *Br J Cancer* **71**: 278–281

Chan R, Rossitto PV, Edwards BF and Cardiff RD (1986) Presence of proteolytically processed keratins in the culture media of MCF-7 cells. *Cancer Res* **46**: 6353–6359

Chou C-F, Smith AJ and Boshr Omary M (1992) Characterization and dynamics of O-linked glycosylation of human cytokeratin 8 and 18. *J Biol Chem* **267**: 3901–3906

Chu Y-W, Runyan RB, Oshima RG and Hendrix MJC (1993) Expression of complete keratin filaments in mouse L cells augments cell migration and invasion. *Proc Natl Acad Sci USA* **90**: 4261–4625

Donald PJ, Cardiff RD, He D and Kendall K (1991) Monoclonal antibody-porphyrin conjugate for head and neck cancer: The possible magic bullet. *Otolaryngol Head Neck Surg* **105**: 781–787

Feizi T, Gooi HC, Childs RA, Picard JK, Uemura K, Loomes LM, Thorpe SJ and Hounsell EF (1984) Tumor-associated and differentiation antigens on the carbohydrate moieties of mucin-type glycoproteins. *Biochem Soc Trans* **12**: 591–596

Garrod DR (1993) Desmosomes and hemidesmosomes. *Curr Opin Cell Biol* **5**: 30–40

Godfroid E, Geuskens M, Dupressoir T, Parent I and Szpirer C (1991) Cytokeratins are exposed on the outer surface of established human mammary carcinoma cells. *J Cell Sci* **99**: 595–607

Haga Y, Horiuchi S, Morino Y and Akagi M (1989) Partial purification and characterization of CA19–9 antigen from the ascitic fluid of a patients with pancreatic cancer. *Clin Biochem* **22**: 363–368

Hanisch FG, Uhlenbruck G and Dienst C (1984) Structure of tumor-associated carbohydrate antigen Ca 19–9 on human seminal plasma glycoproteins from healthy donors. *Eur J Biochem* **144**: 467–473

Hanisch FG, Uhlenbruck G, Dienst C, Stottrop M and Hippauf E (1985) Ca 125 and Ca 19–9: two cancer-associated sialylsaccharide antigens on a mucus glycoprotein from human milk. *Eur J Biochem* **149**: 323–330

Hembrough TA, Vasudevan J, Allietta MM, Glass WF II and Gonias SL (1995) A cytokeratin 8-like protein with plasminogen-binding activity is present on the external surfaces of hepatocytes, HepG2 cells and breast carcinoma cell lines. *J Cell Sci* **108**: 1071–1082

Hembrough TA, Li L and Gonias SL (1996) Cell-surface cytokeratin 8 is the major plasminogen receptor on breast cancer cells and is required for the accelerated activation of cell-associated plaminogen by tissue-type plasminogen activator. *J Biol Chem* **371**: 25684–25691

Hendrix MJ, Seftor EA, Chu YW, Trevor KT and Sftor RE (1996) Role of intermediate filaments in migration, invasion and metastasis. *Cancer Metastasis Rev* **15**: 507–525

Hintner H, Steinert PM and Lawley TJ (1983) Human upper epidermal cytoplasmic antibodies are directed against keratin intermediate filament protein. *J Clin Invest* **72**: 1344–1351

Kalthoff H, Kreiker C, Schmiegel W-H, Greten H and Thiele H-G (1986) Characterization of Ca 19–9 bearing mucins as physiological exocrine pancreatic secretion products. *Cancer Res* **46**: 3605–3607

Klug TL, LeDonne NC, Greber TF and Zurawski VR (1988) Purification and composition of a novel gastrointestinal tumor-associated glycoprotein expressing sialylated lacto-*N*-fucopentaose II (CA 19–9). *Cancer Res* **48**: 1505–1511

Koprowski H, Steplewski Z, Mitchel K, Herlyn M, Herlyn D and Fuhrer P (1979) Colorectal carcinoma antigens detected by hybridoma antibodies. *Somatic Cell Genet* **5**: 957–972

Laemmli UK (1970) Cleavage of structural proteins during the assembly of the head of bacteriophage T4. *Nature* **227**: 680

Modjtahedi N, Frebourg T, Fossar N, Lavialle C, Cremisi C and Brison O (1992) Increased expression of cytokeratin and ferritin-H genes in tumorigenic clones of the SW 613-S human colon carcinoma cell line. *Exp Cell Res* **201**: 74–82

Magnani JL, Steplewski Z, Koprowski H and Ginsburg V (1983) Identification of the gastrointestinal and pancreatic cancer associated antigen selected by monoclonal antibody 19–9 in the sera of patients as a mucin. *Cancer Res* **43**: 5489–5492

Moll R, Franke WW and Schiller DL (1982) The catalog of human cytokeratins: patterns of expression in normal epithelia, tumors and cultured cells. *Cell* **31**: 11–24

Mori M, Mimori K, Inoue H, Bernard GF, Tsuji K, Nanbara S, Ueo H and Ariyoshi T (1995) Detection of cancer micrometastases in lymph nodes by reverse transcriptase-polymerase chain reaction. *Cancer Res* **55**: 3417–3420

Ogawa J, Sano A, Koide S and Shohtsu A (1994) Relation between recurrence and expression of proliferating cell nuclear antigen, sialyl Lewis, and sialyl Lewis in lung cancer. *J Thorac Cardiovasc Surg* **108**: 329–336

Owaribe K, Nishizawa Y and Franke WW (1991) Isolation and characterization of hemidesmosomes from bovine corneal epithelial cells. *Exp Cell Res* **192**: 622–630

Pendleton N, Myskow MW and Green JA (1992) Expression of cytokeratins, involucrin and peanut agglutinin binding lectin in resected non-small cell lung carcinomas. *Br J Cancer* **65**: 37 (abstract)

Pendleton N, Occleston NL, Walshaw MJ, Littler JAH, Jack CIA, Myskow MW and Green JA (1994) Simple cytokeratins in the serum of patients with lung cancer: relationship to cell death. *Eur J Cancer* **30A**: 93–96

Schaafsma HE, Ramaekers FCS, van Muijen GNP, Robben H, Lane EB, Leigh IM, Ooms ECM, Schalken JA, van Moorselaar RJA and Ruiter DJ (1991) Cytokeratin expression patterns in metastatic transitional cell carcinoma of the urinary tract. *Am J Pathol* **139**: 1389–1400

Schaafsma HE, van der Velden LA, Manni JJ, Peters H, Link M, Ruiter DJ and Ramaekers FCS (1993) Increased expression of cytokeratins 8, 18 and vimentin in the invasion front of mucosal squamous cell carcinoma. *J Pathol* **170**: 77–86

Sundstrom B and Stigbrand T (1990) A two site enzyme linked immunosorbent assay for cytokeratin 8. *Int J Cancer* **46**: 604–609

Sugiyama K, Kawai T, Nagata N and Suzuki M (1992) Tumor-associated carbohydrate antigens in primary pulmonary adenocarcinomas and their metastases. *Hum Pathol* **23**: 900–904

Takada A, Ohmori K, Takahashi N, Tsuyuoka K, Yago K, Zenita K, Hasegawa K and Kannagi R (1991) Adhesion of human cancer cells to vascular endothelium mediated by a carbohydrate antigen, sialyl Lewis A. *Biochem Biophys Res Commun* **179**: 713–716

Takada A, Ohmori K, Yoneda T, Tsuyuoka K, Hasegawa A, Kiso M and Kannagi R (1993) Contribution of carbohydrate antigens sialyl Lewis A and sialyl Lewis X to adhesion of human cancer cells to vascular endothelium. *Cancer Res* **53**: 354–361

Towbin H, Staehelin T and Gordon J (1979) Electrophoretic transfer of proteins from polyacrylamide gels to nitrocellulose sheets. *Proc Natl Acad Sci USA* **76**: 4350–4354

*© 1999 Cancer Research Campaign*
